# Optimized laser speckle–based imaging system and methods for deep tissue cerebral blood flow imaging in small rodents

**DOI:** 10.1117/1.NPh.12.2.025017

**Published:** 2025-06-24

**Authors:** Ria Paul, Soumyajit Sarkar, Susweta Das, Shruti D. Marathe, Murali Krishnamoorthy, Nixon M. Abraham, Hari M. Varma

**Affiliations:** a Indian Institute of Technology Bombay (IITB), Department of Biosciences and Bioengineering, Mumbai, Maharashtra, India; b Indian Institute of Science Education and Research (IISER), Laboratory of Neural Circuits and Behaviour (LNCB), Department of Biology, Pune, Maharashtra, India

**Keywords:** laser speckle contrast imaging, multi-exposure speckle imaging, multi-speckle diffuse correlation tomography, cerebral blood flow, olfactory stimulation, forepaw stimulation, preclinical studies, rodents, imaging system

## Abstract

**Significance:**

The imaging of cerebral blood flow in small rodents is crucial for a better understanding of brain functions in healthy and diseased conditions. Existing methods often struggle to provide both superficial and deep tissue blood flow measurements in a non-invasive, flexible, and reliable manner, creating a need for an integrated platform that addresses these limitations.

**Aim:**

We aim to design and develop a multi-modal laser speckle–based imaging platform and associated algorithms to image superficial and deep tissue cerebral blood flow in small rodents.

**Approach:**

A modular design has been adopted to integrate laser speckle contrast imaging and multi-speckle diffuse correlation tomography to a single cerebral blood flow imaging platform for small rodents with an independent module for animal holding and handling. A topographic imaging method, equipped with a filter to remove surface artifacts, was incorporated to image cerebral blood flow changes in response to forepaw and olfactory stimuli activations, with the skull and scalp kept intact.

**Results:**

A significant increase in blood flow was found in the olfactory bulbs of mice post-stimulation by various odors (p<0.01). Similarly, forepaw stimulation resulted in a significant increase in blood flow in the contralateral side of the somatosensory cortex with the application of the filter for skull and scalp intact, skull intact, and skull removed cases (p<0.01).

**Conclusions:**

We have validated our system through functional studies, demonstrating its capability to detect enhanced blood flow changes across the olfactory bulbs and somatosensory cortex in rodents with potential for broad applications in preclinical research.

## 1 Introduction

Cerebral blood flow (CBF) is a vital component of brain function, delivering oxygen and nutrients necessary for proper neuronal activity. Monitoring change in CBF holds significant importance across diverse fields such as neuroscience, cognitive psychology, and clinical research.^1^^–^^3^ One of the major focuses in neuroimaging is to image CBF changes in response to certain functional stimuli.^4^ Although numerous methods exist for measuring CBF, optical imaging methods present distinct advantages particularly in functional activation studies. These methods are non-invasive, radiation-free, and offer high spatial and temporal resolution, making them ideal for observing blood flow changes.^5^ Notable methods include functional Near-Infrared Spectroscopy (fNIRS),^6^ Diffuse Optical Spectroscopy/Tomography (DOS/T),^7^ Diffuse Correlation Spectroscopy/Tomography (DCS/T),^8^ Speckle Contrast Optical Spectroscopy/Tomography (SCOS/T),^9^ and Laser Speckle Contrast Imaging (LSCI)^10^ which provide valuable insights into physiological processes.

Multipronged experimental approaches using animal models based on clinical observations are often required to understand the underlying neural mechanisms of brain dysfunctions.^11^^–^^16^ Among these, preclinical imaging is often deployed but not limited to understanding pathophysiology and drug testing.^17^^–^^20^ It is also used to test the effectiveness and safety of imaging modalities for translation studies. In the abovementioned context, small animal imaging platforms are crucial for visualizing and monitoring biological processes in laboratory animals such as mice and rats. To address consistency and reproducibility challenges in existing platforms, it is essential to integrate various optical modalities into a modular small-animal imaging platform that is flexible, customizable, and scalable to meet diverse research needs.

We have recently introduced a camera-based high-density diffuse correlation spectroscopy system called multi-speckle diffuse correlation spectroscopy^21^^,^^22^ and its tomography counterpart which is multi-speckle diffuse correlation tomography (M-DCT)^23^ which were validated by measuring CBF in healthy humans. We present a modular design–based small-animal imaging platform integrating LSCI and M-DCT modalities. The integration of various laser speckle imaging methods will provide complementary information enabling simultaneous understanding of biological structures and functions. The system can be broadly divided into two detachable modules, one being the top module containing the scanning laser source and measurement optics, whereas the bottom module consists of a modified stereotaxic frame to hold and handle both mice and rats. We have also made three-axis maneuvering of optics sub-modules such as laser source, galvo mirror scanners, camera, and the stereotaxic frame for better focusing and scanning of the brain region.

We further integrate a novel topographic imaging algorithm equipped with a filter to the M-DCT for fast and almost real-time CBF imaging in skull- and scalp-intact mice and rats. The preservation of the scalp and skull during functional studies enhances animal survival rates, facilitating longitudinal studies with longer time courses. We validate this platform by measuring relative changes in cerebral blood flow (rCBF) during forepaw stimulation in mice and rats, utilizing surface topography imaging.

Furthermore, we extend the application of our platform to investigate the effects of olfactory stimulation on CBF dynamics in mice. Recent advancements in olfactory imaging studies in mice have revealed intricate neural processing mechanisms. Calcium imaging^11^^,^^24^^–^^29^ and functional magnetic resonance imaging (fMRI)^30^ have been used to investigate olfactory bulb functions and neural responses to olfactory stimuli in rodents. Optical intrinsic signal imaging,^31^^–^^33^ electroencephalography,^34^ and electrocorticography^35^ are some other techniques for studying neural function in olfactory stimulations. Although these techniques can be used to study neuronal representations, a reliable method for investigating hemodynamics in the brain and its correlation with neural activity at the population level is missing. Our results report the first extensive attempt toward this using an array of odorants and by analyzing the evoked responses in mice employing laser speckle technologies. Mice have been given odors belonging to different chemical groups to show blood flow dynamics over the olfactory bulb (OB). Although one previous study^36^ has shown preliminary results of olfactory stimulation by LSCI using specific odors, we explore the impact of several odors from different chemical families over a larger region of interest (ROI) and for a longer duration. This is achieved by a novel topographic scanning algorithm to measure spatio-temporal blood flow changes. We observed odor-specific hemodynamics in the main OB, the first relay station in olfactory information processing. This comprehensive method therefore provides a promising tool for understanding neurovascular coupling.

## 2 Methods and Materials

### 2.1 Imaging Platform: Design and Features

We propose a generic small-animal imaging platform designed to measure deep tissue and superficial blood flow, as well as oxy- and deoxy-hemoglobin (${\mathrm{HbO}}_{2}$ and Hb, respectively) concentration. In the current study, however, we have restricted to speckle-based blood flow imaging and have not demonstrated dual-wavelength Near-Infrared Spectroscopy (NIRS)-based measurement of oxygen saturation. The platform combines various laser imaging methods such as LSCI, DOT, and DCT, each operating independently. A graphical user interface (GUI) controlled by a single-board computer (SBC) (5), Raspberry Pi 4B, facilitates interaction between different modules and real-time monitoring of animal health which is displayed on a screen (12). In this paper, we demonstrate the setup for measuring relative changes in blood flow using M-DCT for deep tissue blood flow and LSCI for surface blood flow. The GUI helps in choosing the type of imaging, whether LSCI or scanning, as well as selecting imaging parameters and live monitoring of the animal.

**Fig. 1:**
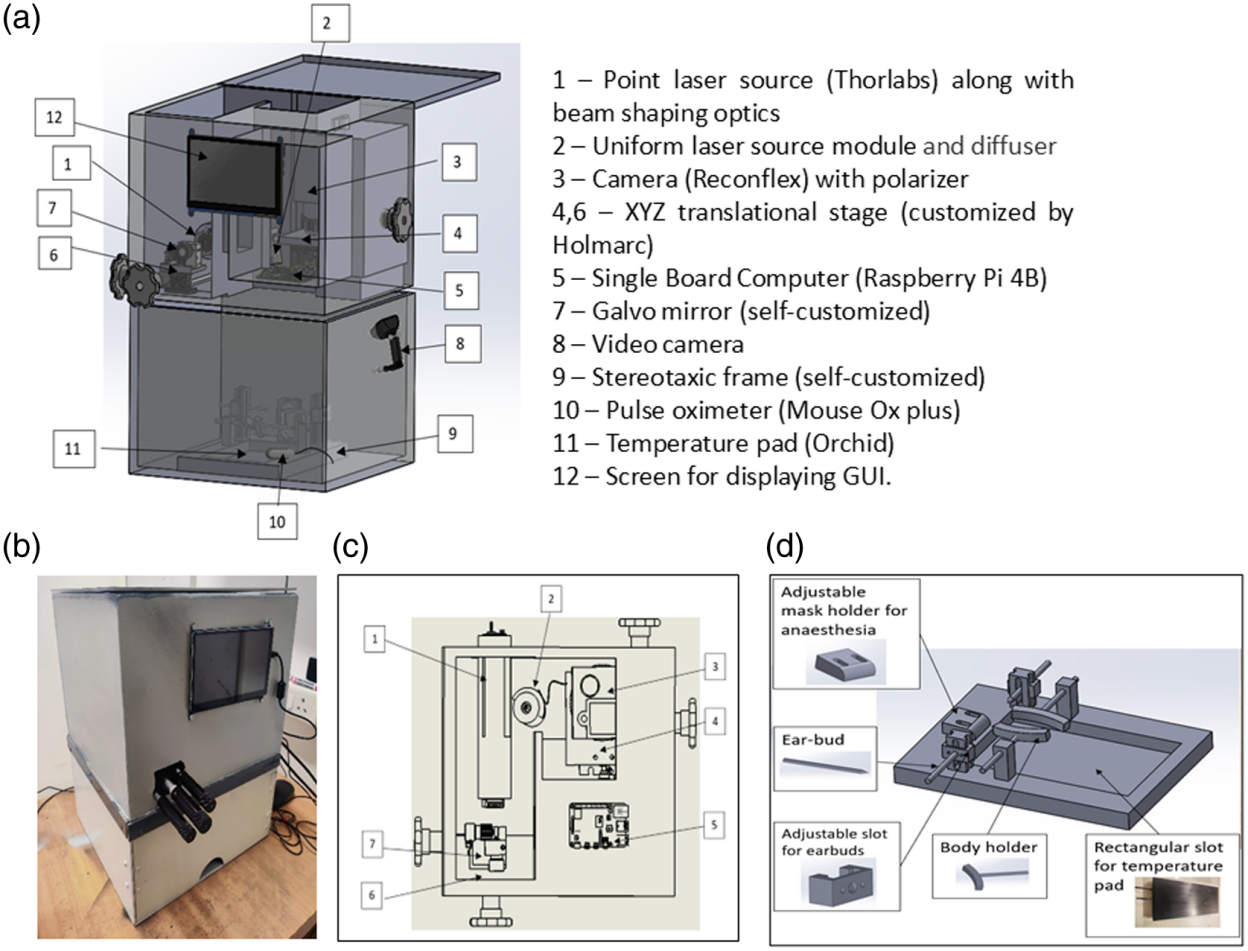
Overview of the modular imaging system. (a) Design of the proposed modular imaging system along with the individual modules as specified in the adjacent right panel. (b) Assembled modular imaging system. (c) Top module comprising different optics such as laser sources, galvo, camera, and SBC. (d) Components of the bottom module, featuring the stereotaxic frame utilized for securing the animal, alongside probes for monitoring the health status of the animals.

The imaging platform is broadly divided into top and bottom modules as shown in Fig. 1(a). The top module, as shown in Fig. 1(c), comprises a point laser source (1) (λ=785  nm, Thorlabs LDM785, Newton, New Jersey, United States), a uniform source (2) (λ=785  nm, laser diode—Thorlabs L785P090) with a diffuser, a dual-axis galvo mirror (7, self-customized), and a camera (3) (ReconFlex 1920). Precise adjustments of the camera and galvo mirror positions are facilitated by two XYZ translational stages (4, 6) (Holmarc, Kochi, India), which enable focusing the ROI onto an image plane keeping the movement of the animal to a minimum. The imaging was performed using a 35-mm fixed focal length objective lens (Edmund Optics, Barrington, New Jersey, United States) along with tubes of different lengths for different studies. A MouseOx^®^ Plus pulse oximeter (Starr Life Sciences, Oakmont, Pennsylvania, United States) was used for monitoring physiological parameters. To maintain body temperature during experiments, a rodent thermal pad (Orchid Scientific, Nashik, India) was used. Moreover, all optics modules are designed to be easily replaceable or upgradable based on user requirements and market availability. The image of the assembled modular imaging system is shown in Fig. 1(b). The system provides finer focusing and scanning of the ROI in the rodent brain while accounting for its curvature with the help of three-dimensional maneuvering of laser source and scanning optics. The bottom module housing the animal includes an adjustable customized stereotaxic frame (9) and provisions for an anesthesia system, exhaust fan, pulse oximeter (10), temperature pad (11), video camera (8), and illuminating white lights. Our customizable stereotaxic frame, as shown in Fig. 1(d), features movable earbars, body-holding structures, and a mask holder, facilitating the accommodation of various breeds of rodents. The stereotaxic frame in the lower module is attached to an XYZ translation stage for fine adjustments.

### 2.2 Theory and Algorithms

#### 2.2.1 Brief theory of M-DCT and proposed filter design

M-DCT makes use of the Volterra integral equation form of the relation connecting multi-exposure speckle contrast to field autocorrelation ($g_{1}(\tau )$) which is given by

$$\kappa (T)=\frac{2\beta }{T}\int_{0}^{T}g_{1}(\tau {)}^{2}(1-\frac{\tau }{T})d\tau ,$$ (1)

where T is the exposure time of the camera, and τ is the correlation delay time. As explained in Refs. 21 and 23, $g_{1}(\tau )$ is obtained from the correlation diffusion equation (CDE) which models the diffusion of autocorrelation of the electric field of light in a dynamic turbid medium such as tissue.^7^ M-DCT utilizes the algorithm termed as multi-step Volterra integral method^21^ to extract $g_{1}(\tau )$ from κ(T). This has the advantage of replacing otherwise expensive DCS detectors with less expensive cameras but with minimal loss of information.^37^ We further apply a filter to $g_{1}$ to filter out the surface artifacts and extracerebral interferences which helps to achieve better deep tissue imaging with the skull and scalp intact.

For a given source and detector position $r_{s}$ and $r_{d}$, respectively, the filtered autocorrelation in the Fourier domain is given by

$${\overset{˜}{g}}_{1}^{F}(r_{s},r_{d},\omega )={\overset{˜}{g}}_{1}(r_{s},r_{d},\omega )\times \overset{˜}{F}(\omega ).$$ (2)

Here, ${\tilde{g}}_{1}(r_{s},r_{d},\omega )$ is the Fourier transform (FT) of $g_{1}(r_{s},r_{d},\tau )$. The filter is defined as $\overset{˜}{F}(\omega )=1-Q(\omega )/\max(Q(\omega ))+\epsilon$, where $Q(\omega )\equiv \mathrm{FT}(e^{-k\sqrt{\tau }})$ and ϵ is a constant shift. Although we have introduced this filter for M-DCT in humans,^23^ here, we fine-tune the filter parameters to adapt it for animal brain imaging. The filter is designed based on the frequency spectrum of field auto-correlation measurement taken from cerebral blood flow in rodents. We define $S\equiv \frac{\partial }{\partial r}\;\max_{\omega }|\Delta {\overset{˜}{g}}_{1}(r_{s},r_{d},\omega )|$ for different source-detector (SD) separations (r), where $\Delta {\tilde{g}}_{1}(r_{s},r_{d},\omega )$ is the FT of the perturbation in measurement.^23^ We define an optimal SD separation (${\mathrm{SD}}_{\mathrm{opt}}$) by choosing the r at which S has the first abrupt change. We use prior information on the thickness of the scalp and skull, with specific values assigned for mice (450  μm)^38^ and rats (750  μm)^38^^,^^39^ to simulate and find the ${\mathrm{SD}}_{\mathrm{opt}}$ at which the sensitivity shows the first abrupt change. We select 1.2 and 1.9 mm as ${\mathrm{SD}}_{\mathrm{opt}}$ from our simulation for mice and rats, respectively, when the skull is present. In case of post-skull removal, we use ${\mathrm{SD}}_{\mathrm{opt}}$ of 1 and 1.3 mm in mice and rats, respectively. It should be noted that ϵ ranging from 1.5 to 1.9^23^ gives relatively less error. In this paper, we have used ϵ=1.5 in the design of a filter for the proposed method. The filter is applied separately to both baseline and stimulation measurement data to facilitate M-DCT and subsequent topographic imaging.

#### 2.2.2 Topographic scanning algorithm

**Fig. 2:**
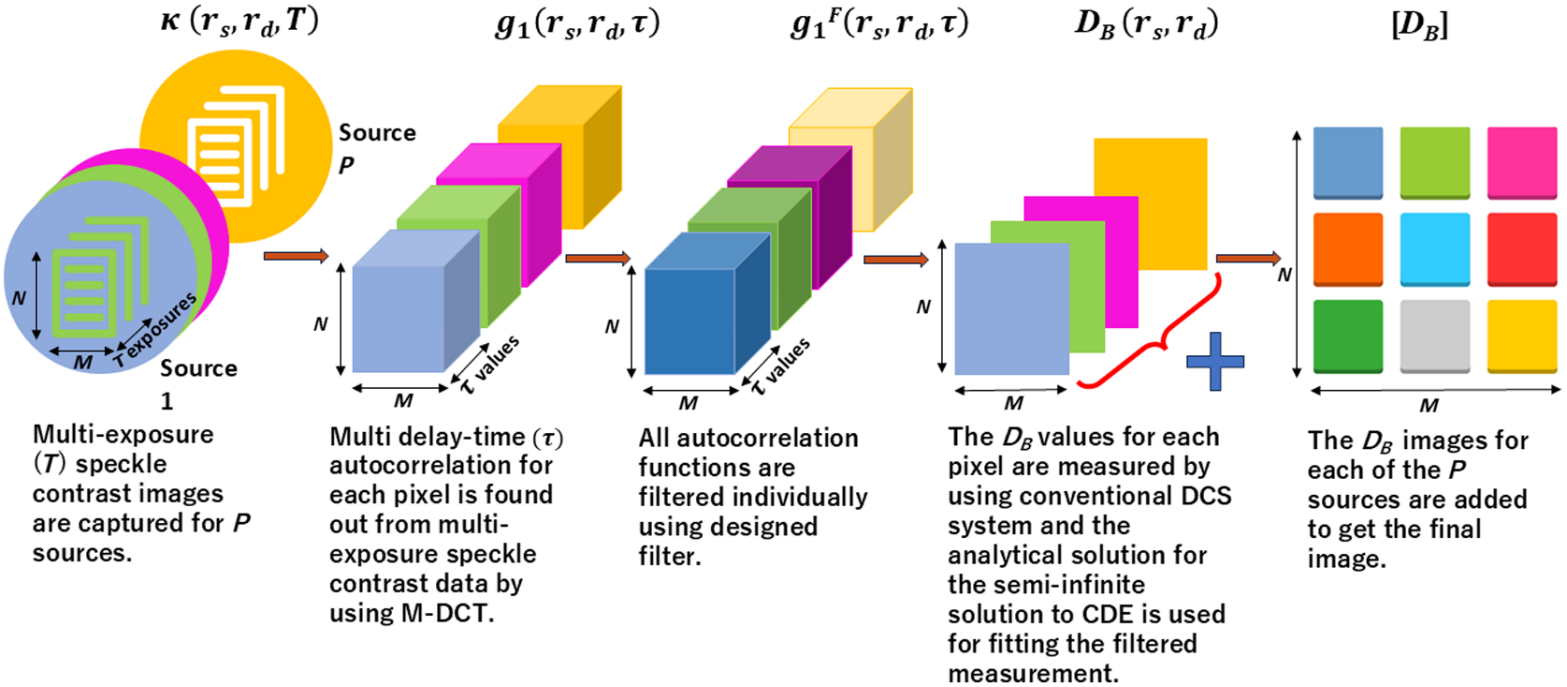
Schematic for performing topographic scan along with filter method.

Instead of a full-fledged image reconstruction done in our earlier work^23^ or approaches without inversion^40^ and similar other works,^41^^–^^44^ we introduce a simple topographic imaging algorithm as explained below. The inverse Fourier transform of the filtered auto-correlation from Eq. (2) as a function of τ is fitted to the semi-infinite domain solution of CDE^5^ for determining the particle diffusion coefficient $D_{B}$. The parameter $D_{B}$ is proportional to the blood flow.^5^ We repeat the fitting for $D_{B}$ for every detector position thus comprising the CBF image for a given source position in the topography scan, $D_{B}^{i}$, i=a or b, where a corresponds to the baseline and b corresponds to the perturbation values. We perform a summation of all such images from multiple source positions from the entire scan to get the final image of CBF, $\left[D_{B}^{i}\right]$. We repeat the above procedure for both the baseline matrix, [$D_{B}^{a}$], and perturbed measurement matrix, [$D_{B}^{b}$], during a functional activation. The final image in the topographic scan is obtained by subtracting the baseline CBF images from the perturbed CBF measurement ($\left[D_{B}^{b}]-[D_{B}^{a}\right]$) and subsequently applying a moving average filter. The entire process of generating autocorrelation from multi-exposure speckle measurement using M-DCT, filtering, and topography algorithm is shown in Fig. 2.

#### 2.2.3 Data processing strategy

The multi-exposure speckle imaging (MESI) data are obtained at 10 exposure times between 30  μs and 3 ms using a uniform laser source. The raw intensity data acquired are processed to obtain the speckle contrast at every pixel temporally. To obtain the rCBF images from LSCI, the experimental multi-exposure data were fitted to the model given in Eq. (3) to obtain $\tau_{c}$. After finding the $\tau_{c}$ images for both baseline and functional stimulation, we normalized the images with respect to the baseline to produce the rCBF image. For topographic blood flow imaging, a laser source incident on a custom-made galvo mirror was focused to a point of beam diameter 0.9 mm of 5 mW power and was used to scan over nine source points on the brain during baseline and functional stimulation of the olfactory bulbs and cortical region, respectively. A Complementary Metal-Oxide-Semiconductor (CMOS) camera (Reconflex) with 1920×1440 resolution and 234 fps was used to capture intensity images. The image comprises 800×640 pixels covering a field of view (FOV) of 4  mm×3.5  mm for olfactory stimulation and 1400×1400  pixels over an FOV of 8.5  mm×8.5  mm for forepaw stimulations in mice. The image comprises 1400×1400  pixels covering an FOV of 12  mm×12  mm for forepaw stimulations in rats. For every exposure, 200 images were used for processing the data using M-DCT and LSCI.

For LSCI during olfactory stimulation, the inverse correlation time (ICT) and the velocity of blood flow (v) are computed based on the correlation decay time ($\tau_{c}$). The decay time is obtained from the speckle contrast data by fitting it to the following equation:^45^^,^^46^

$$\kappa^{2}=\beta (\frac{\tau_{c}}{T}+\frac{\tau_{c}^{2}}{T^{2}}(e^{-\frac{2\;T}{\tau_{c}}}-1)),$$ (3)

from which the velocity of blood flow is obtained as Refs. 45, 47, and 48 $v=\frac{\lambda }{2\pi \tau_{c}}$, where λ is the wavelength of the light (λ=785  nm). The value of β was found to be 0.25 using a solid polydimethylsiloxane phantom and was assumed constant throughout the experiment. The rCBF during olfactory stimulation is defined as

$$\mathrm{rCBF}\%=\frac{v_{b}}{v_{a}}\times 100\%,$$ (4)

where $v_{a}$ represents the mean baseline velocity, and $v_{b}$ is the mean velocity during olfactory stimulation. We use either single exposure or MESI data to fit for $\tau_{c}$ using Eqs. (3) and (4). Similarly, the deep tissue rCBF for forepaw stimulation is defined as

$$\mathrm{rCBF}\%=\frac{D_{B}^{b}}{D_{B}^{a}}\times 100\%,$$ (5)

where $D_{B}^{a}$ and $D_{B}^{b}$ are the mean particle diffusion coefficients for the baseline and forepaw stimulation, respectively.

### 2.3 Animals and Study Design

The experimental procedures used in this paper are approved by the Institutional Animal Ethics Committee (IAEC) at IISER Pune, and the Committee for the Control and Supervision of Experiments on Animals (CCSEA), Government of India (animal facility CCSEA registration number 1496/GO/ReBi/S/11/CCSEA). The usage of animals in this paper was approved under protocol number IISER-Pune/IAEC/2019-2/04. A total of 14 animals were included in this study, comprising 11 mice and 3 rats. Olfactory stimulation experiments for the LSCI study were conducted on five mice, whereas three mice were utilized for olfactory stimulation in the topographic scanning algorithm investigation. For the forepaw stimulation study, three mice and three rats were used.

#### 2.3.1 Olfactory stimulation

Five C57BL/6 mice, aged between 4 and 5 months and weighing 20 to 25 g, were included in this study. The mice were anesthetized with a ketamine and xylazine mixture in a 3:1 ratio for olfactory studies using LSCI. They were exposed to various odors (>99% purity, Sigma Aldrich, St. Louis, Missouri, United States), categorized broadly into esters: isoamyl acetate (AA) and ethyl butyrate (EB); aldehydes: hexanal (Hex) and octanal; phenylpropanoids: eugenol and cineole (Cin); and terpenoids: carvone+ (C+) and carvone− (C−), with water used as a control to demonstrate its negligible impact on change in blood flow.

Each odor was administered for 10 s following a 60-s baseline, with data recorded for 180 s for each odor to assess response time separately. After every odor stimulation, the exhaust fan was activated before the next odor, and sufficient rest was given between two consecutive odor stimulations. All studies were repeated three times in each animal. The $v_{a}$, as defined in Eq. (4), was determined during the first 50 s of the baseline period, whereas $v_{b}$ was determined over 50 s after exposure to the odor, i.e., 60 to 110 s. We computed the FT of κ(t) as $\tilde{\kappa }(f)=\mathrm{FT}(\kappa (t))$ to quantify the rate of blood flow changes in the concerned blood vessel of the olfactory bulb of the animal for both baseline and odor stimulation data, as explained above.

A spatio-temporal ICT image series over the olfactory bulb for 60 s, with each image displayed after 1 s for a single exposure value was also recorded. The olfactory stimulation was initiated using AA. The ICT images were generated from the single exposure data using Eq. (3).

#### 2.3.2 Forepaw stimulation

Three C57BL/6 mice, aged 4 to 5 months and weighing 25 to 30 g, were anesthetized with ketamine and xylazine for forepaw stimulation studies^49^ in the somatosensory cortex. The experimental protocol involved measuring data at three stages: with the skull and scalp intact, with the scalp removed, and with both the skull and scalp removed. Forepaw stimulation was administered to the right forepaw using a pulse of 1-mA current at 4-Hz frequency with a 200-ms pulse width for 15 s, followed by a 45-s rest period^49^ by the current stimulator (Digitimer DS3). The experiment was conducted under three conditions: (a) scalp and skull intact, (b) scalp removed, and (c) skull removed over the cortical region, with each stage repeated three times per animal.

In addition, three Wister rats, aged 2 to 3 months and weighing 280 to 300 g, were used for forepaw stimulation studies over the cortical region. Stimulation involved a pulse of 1.5-mA current at 6-Hz frequency with a 200-ms pulse width for 30 s, followed by a 90-s rest period.^50^ Data were collected under two conditions: (a) scalp and skull intact and (b) scalp removed.

### 2.4 Statistics

Based on the existing literature using forepaw stimulation in rodents,^51^ we anticipate an average change in rCBF of ∼38% with a standard deviation of 9%. With these parameters, we can achieve over 85% statistical power at a significance level (α) of less than 0.001 with a sample size of three rodents. The experiments were not randomized, and the authors were not blinded to allocation during experiments and outcome assessment. Statistical analysis of the rCBF values was conducted using paired or unpaired t-tests to determine significance (p-value). To test significance in more than two groups, one-way Analysis of Variance (ANOVA) was used. p-values less than 0.05 were deemed significant (*p<0.05 and **p<0.01).

## 3 Results

### 3.1 Blood Flow Imaging Using LSCI During Olfactory Stimulation in Mice

**Fig. 3:**
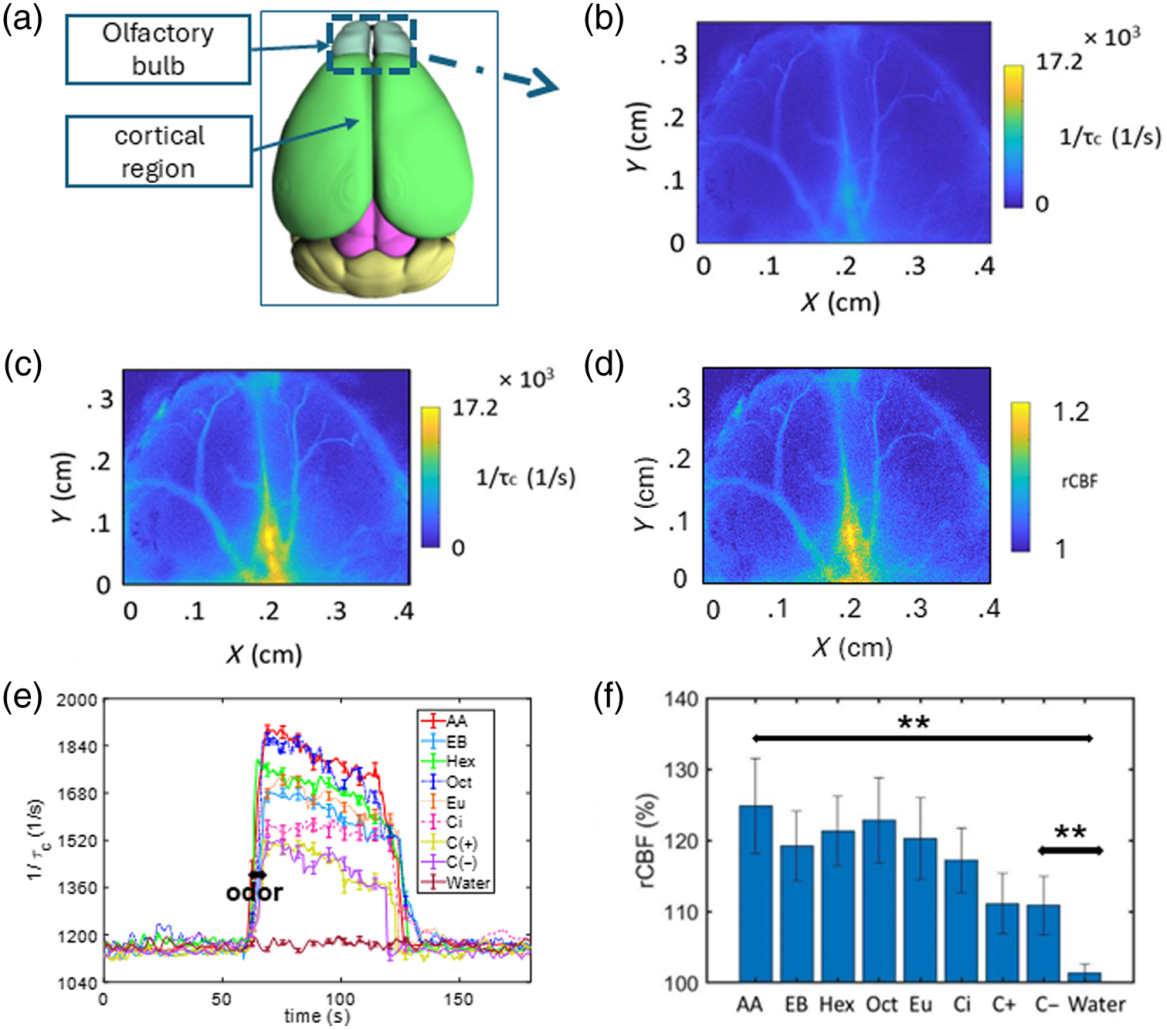
CBF responses to olfactory stimulation in mice. (a) Representation of the mouse brain highlighting the OB and cortical areas imaged (image adapted and modified from the Allen Brain Atlas^52^). (b) ICT image using MESI data obtained from the OB during baseline conditions. (c) ICT image using MESI data obtained from the OB during olfactory stimulation. (d) rCBF observed in the OB during exposure to odors using MESI data. (e) Aggregated data showing the mean and variance in ICT obtained from single-exposure LSCI, shown in terms of percentage, for three trials in an individual mouse subjected to olfactory stimulations using multiple chemicals. (f) Mean rCBF during odor stimulation across five mice.

The representation of a mouse brain indicating the cortical and olfactory bulb is shown in Fig. 3(a). After the removal of the scalp and skull over the olfactory bulb, the ICT and rCBF of the measured data using MESI data as described in Sec. 2.2.3 are shown in Figs. 3(b)–3(d). Figures 3(b) and 3(c) show the ICT during baseline and olfactory stimulation with AA, respectively, whereas Fig. 3(d) shows the corresponding rCBF values. Figure 3(e) shows the ICT of the surface blood flow as a function of time using single-exposure LSCI for multiple odors. It shows that the blood flow increases after exposure to the odor and returns to baseline after ∼60 to 80 s for all odors. The mean and variance are plotted by administering the odors to a given mouse three times. Figure 3(f) shows the mean rCBF (%) corresponding to five mice over a small ROI in the olfactory bulb for different odors. Notably, AA odor, an ester, exhibited a significantly higher rCBF, whereas C−, a monoterpene, showed a comparatively smaller rCBF. Nonetheless, even the small change is statistically significant (p<0.01) when compared with the control group (water). A one-way ANOVA revealed a significant difference in the rCBF responses to odor stimulation, F(8,72)=259.73, p<0.0001. A post hoc analysis using the Games Howell test revealed a significant difference in the rCBF responses of each odor compared with water and also a significant difference among various odors as well.

**Fig. 4:**
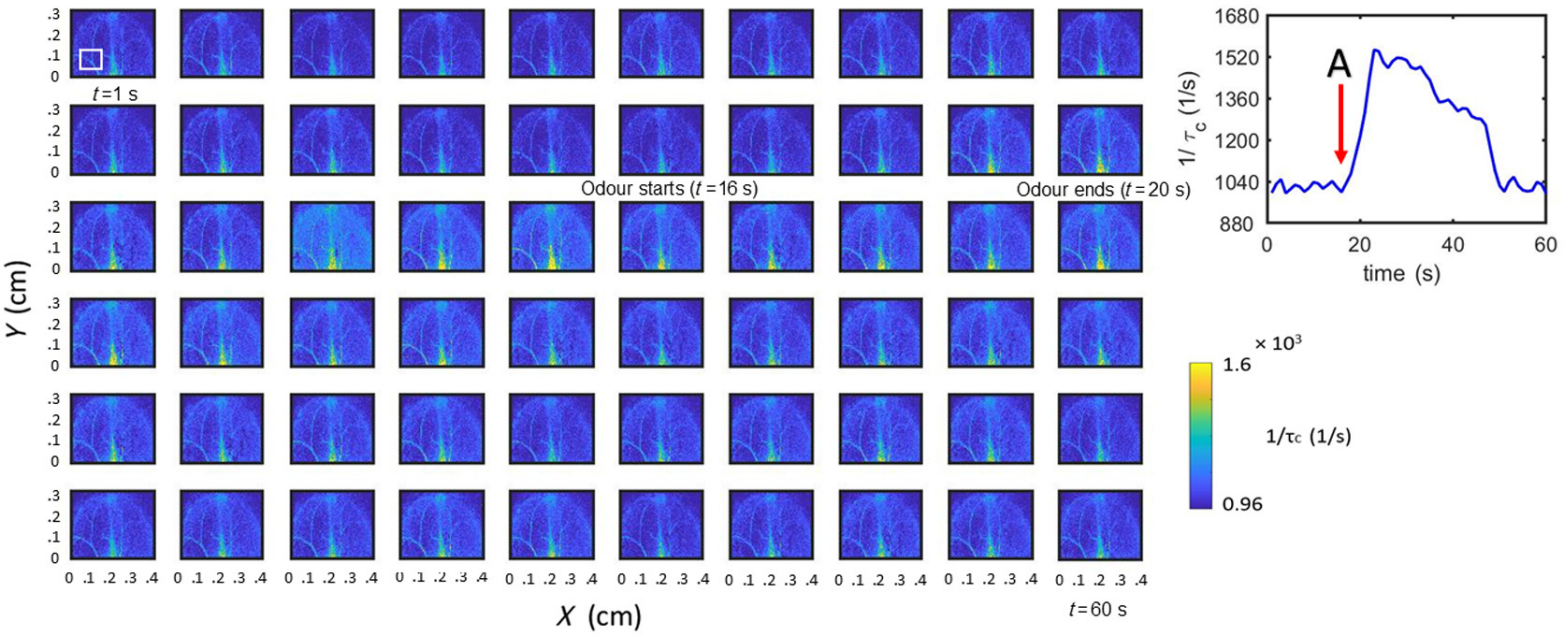
Spatio-temporal series plot of ICT obtained from single-exposure LSCI for olfactory stimulation over 60 s for a mouse (with the skull removed) along with the mean ICT plot over a small window of 0.1  cm×0.1  cm as marked by a white square. A denotes the time at which odor is given (shown in the image series and the ICT plot).

In Fig. 4, we have shown the spatio-temporal blood flow changes in mice in response to odor stimulation. A series of spatio-temporal ICT images over the olfactory bulb were acquired for 1 s each at a single exposure of 1.5 ms over a time duration of 60 s. At 16 s, the olfactory stimulation was given using AA for a duration of 5 s. This series captured both the blood flow changes and its location in response to stimuli. The ICT image shown on the right shows the mean blood flow changes in response to the olfactory stimulus, over the vessels within the white box of size 0.1  cm×0.1  cm, as shown in the first image of the series.

**Fig. 5:**
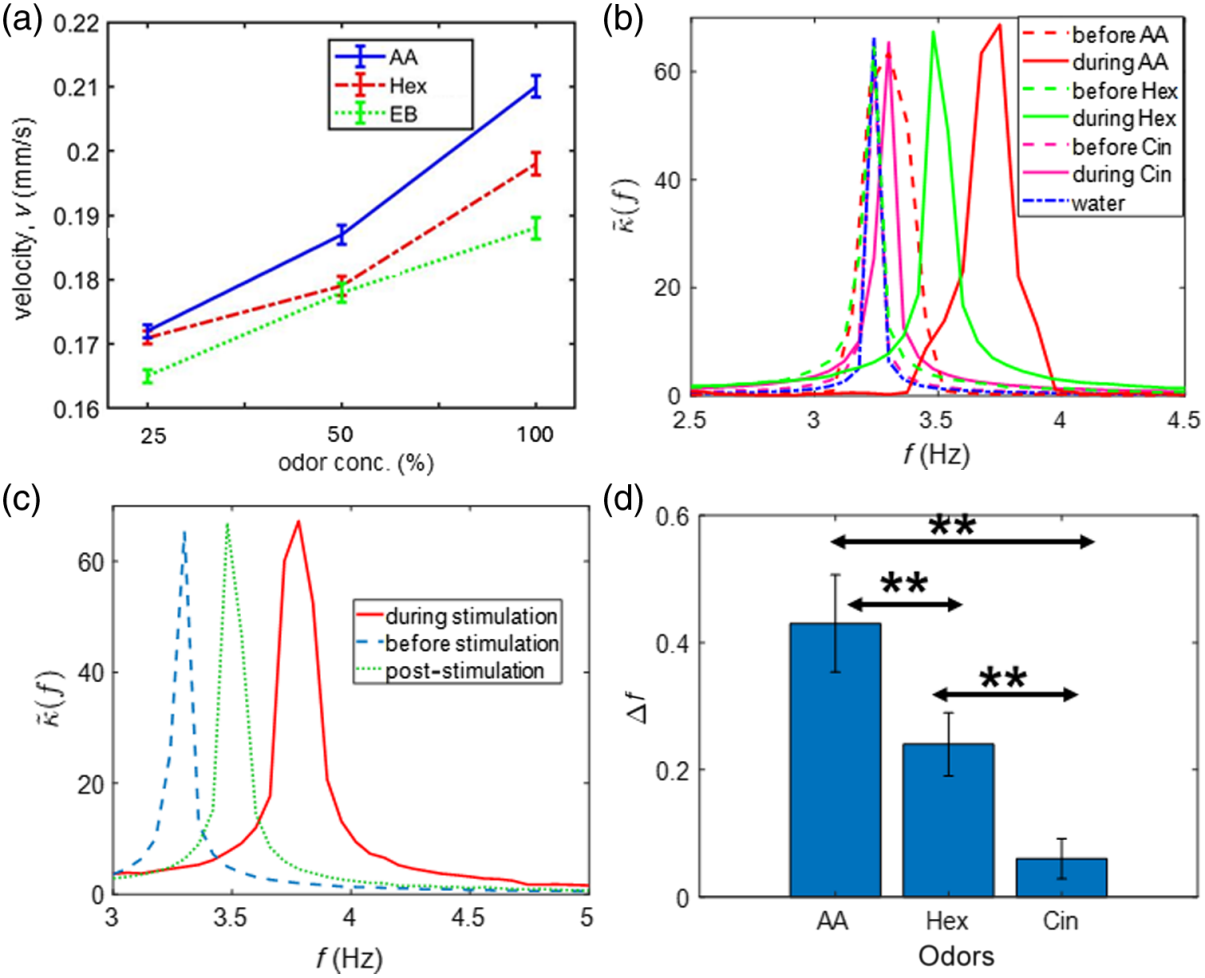
Blood flow changes corresponding to different odors during olfactory stimulation. (a) Increase in blood flow velocity measured at various concentrations of the odors in all mice. (b) Alteration in the frequency of blood flow velocity preceding and succeeding odor stimulation by AA, Hex, Cin, and water for all mice. (c) Same for AA corresponding to before (blue), during (red), and post-odor stimulation (green) in three trials of one mouse. (d) Frequency shift in blood flow following odor stimulation by these odors in all mice.

In Fig. 5, the blood flow changes in response to various odors is shown. Odors belonging to different chemical classes showed varying velocities of blood flow. In addition, three odors (AA, Hex, and EB) were used in different concentrations (25%, 50%, and 100%) in a mineral oil solution to study the effect on blood flow velocity in five mice. For all odors, the blood flow increased with the concentration of odors, as shown in Fig. 5(a). In addition to changes in blood flow, Fig. 5(b) shows the alterations or shifts in the frequency of blood flow velocity before and during odor stimulation by AA, Hex, and Cin in all mice (n=5). It can be seen that the shift decreases from AA to Cin, and for water, no shift was observed. Figure 5(c) shows the same corresponding to before, during, and after stimulation by AA in one mouse for three trials. This was obtained by performing an FT on three segments of the blood flow velocity data—before stimulation (before odor administration), during stimulation (including odor stimulation part until the blood flow remains above baseline), and post-stimulation (where the blood flow returns to its initial baseline value). It can be seen that the post-stimulation peak shifts toward the value of before stimulation peak. This indicates that the frequency shift is in fact due to the odor stimulation as the blood flow also returns to its baseline value as seen in Fig. 3(c). A quantification of the frequency shifts (Δf) observed for the three odors across all mice has been shown in Fig. 5(d). A one-way ANOVA revealed a significant difference in the frequency shifts due to odor stimulation, F(2,12)=55.2, p<0.0001. A post hoc analysis using the Games Howell test revealed a significant difference in the frequency shifts among the three odors.

### 3.2 Topographic Imaging in Mice During Olfactory Stimulation

**Fig. 6:**
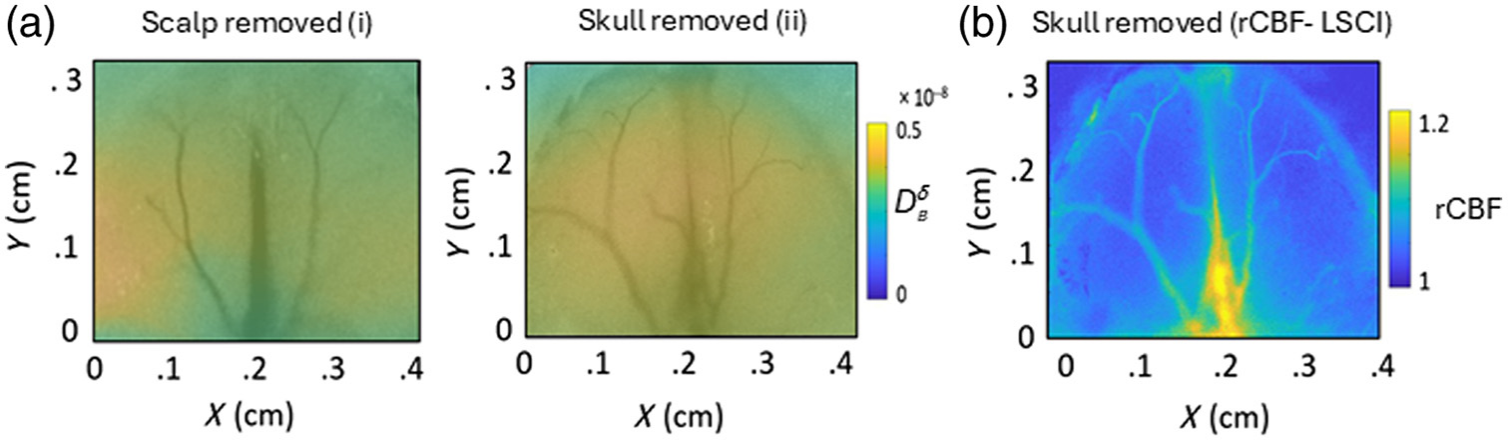
Topographic imaging during olfactory stimulation on a mouse. (a) Topographic image superimposed on white light image over olfactory region with scalp removed (i) and skull removed (ii). (b) rCBF from MESI image before and after odor stimulation for the skull removed case.

The filtered topographic measurement image is superimposed on a white light image for skull-intact and skull-removed mice over the olfactory region as shown in Fig. 6(a). The rCBF image, obtained from MESI, before and after odor stimulation with the scalp and skull removed as shown in Fig. 6(b) confirms that the change in blood flow due to odor stimulation happened in both the lobes as shown by the topographic study.

### 3.3 Blood Flow Changes Associated with Forepaw Stimulation in Mice

**Fig. 7:**
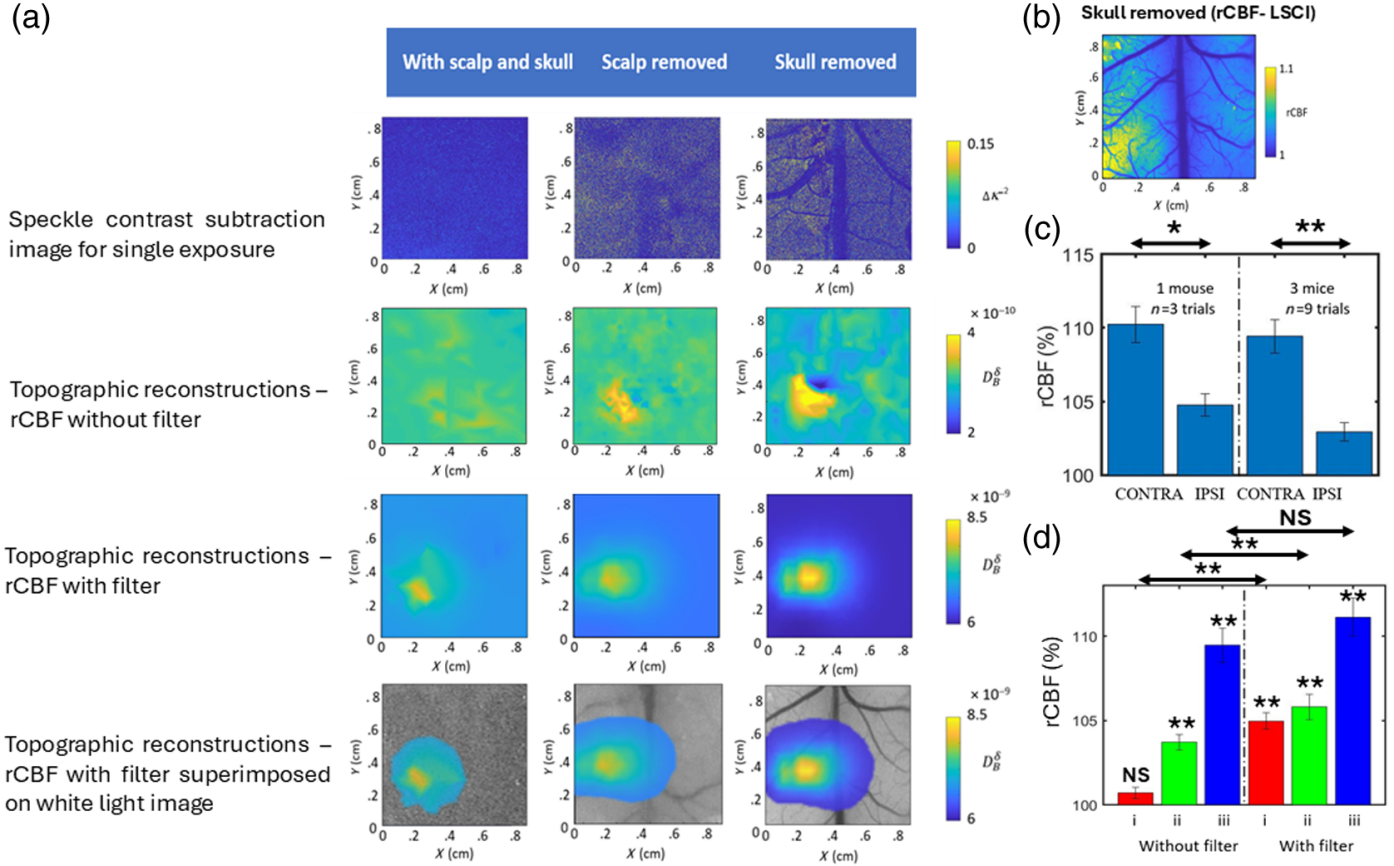
CBF during forepaw stimulation in mice. (a) Topographic images with and without a filter, showing three anatomical stages: intact scalp and skull, scalp removed, and skull removed, along with speckle contrast image. (b) rCBF measurement from MESI with the skull removed demonstrating increased blood flow in the left hemisphere during right forepaw stimulation. (c) Comparison of the mean rCBF changes between ipsilateral and contralateral hemispheres without a filter in conventional tomographic reconstruction after removal of the skull. (d) Mean rCBF (%) comparison in a topographic scan with and without filter use, highlighting significant changes even with the presence of the skull. (i) Scalp and skull intact, (ii) scalp removed, and (iii) skull removed. *p<0.05. **p<0.01. NS, not significant.

Figure 7 shows the results of CBF measurements during forepaw stimulation in mice. Figure 7(a) presents topographic images obtained with and without using a filter, showing three anatomical stages: scalp and skull intact, scalp removed, and skull removed. The first row of the figure also shows a simple multi-source subtraction image for a single-exposure LSCI data. In Fig. 7(b), the rCBF image obtained from MESI is shown for a mouse with the skull removed. Notably, despite variations in values, the location of perturbation in the topographic image is comparable to that observed in the rCBF image in the stage where the skull is removed. However, we could not visualize the CBF with the scalp and skull intact without the filter. The location of the perturbation can be seen only when the filter is used in all intact cases. Figure 7(c) presents a comparison of the mean and variance of rCBF between the ipsilateral and contralateral hemispheres, over three trials in three mice without filter for conventional tomographic reconstruction. The asymmetrical response between the ipsilateral and contralateral sides is attributed to the well-known neurovascular coupling associated with forepaw stimulation in mice. Figure 7(d) shows the mean and variance rCBF (%) comparisons in a topographic scan with and without filter use, plotting rCBF for the same three stages (“i”, “ii,” and “iii”). Significant changes (**) in rCBF are seen after the application of the filter for the cases (“i” and “ii”) as seen in the right panel of the figure as compared with the no-filter cases (left panel of the figure). In addition, when we remove the skull and scalp (“iii”), rCBF measurements improve slightly after using the filter, indicating that the filter helps to remove surface artifacts. All these results showcase the efficacy of the filter in showing CBF changes with high statistical significance.

### 3.4 Blood Flow Changes Associated with Forepaw Stimulation in Rats

**Fig. 8:**
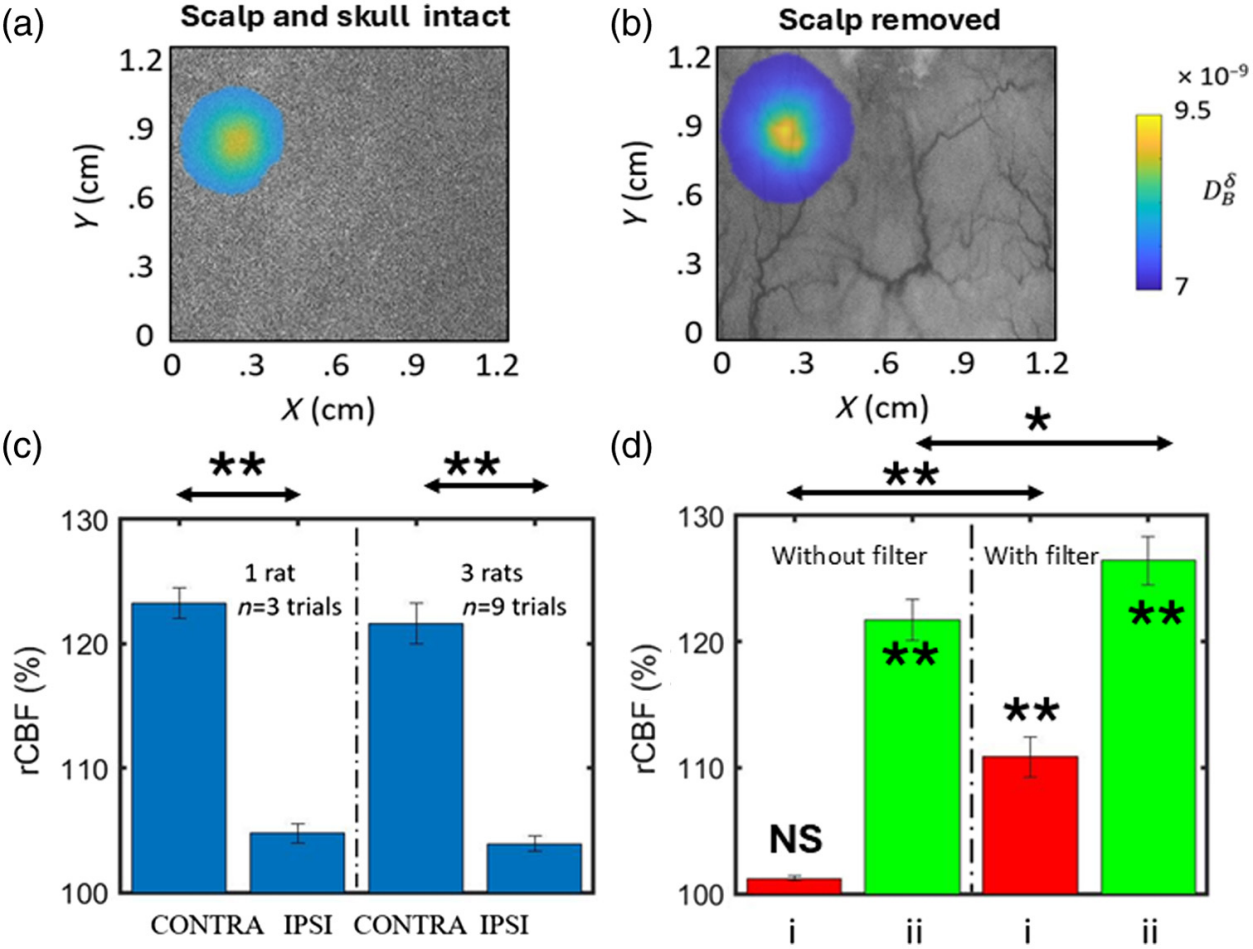
rCBF localization in rat brain during forepaw stimulation. (a) Topographic imaging at the cortical region due to right forepaw stimulation with the scalp and skull intact superimposed on the white light image. (b) With the scalp removed at the same location. (c) Comparison of the mean rCBF changes between ipsilateral and contralateral hemispheres without a filter in conventional tomographic reconstruction after removal of the scalp. (d) Mean rCBF (%) comparison in a topographic scan with and without filter use, highlighting significant changes even with the presence of the skull. (i) Intact scalp and skull and (ii) scalp removed. *p<0.05. **p<0.01. NS, not significant.

In a similar study conducted on rats with forepaw stimulation, the rCBF is shown in Fig. 8. Figure 8(a) illustrates the filtered rCBF data obtained from topographic scanning at the cortical region in response to right forepaw stimulation, with both the scalp and skull intact. Subsequently, Fig. 8(b) shows the same region with the scalp removed, emphasizing the filter’s role in rCBF reconstruction under both conditions. The consistent localization of rCBF signals, with and without the scalp, is attributed to the usage of the filter. In Fig. 8(c), the mean rCBF on the ipsilateral and contralateral sides for one and three rats with scalp removed during right forepaw stimulation is quantified by conventional tomographic reconstruction. The results reveal a significant increase in blood flow on the contralateral side, whereas the increase in blood flow on the ipsilateral side is not significant.

Figure 8(d) provides rCBF quantification for topographic scans with and without the application of the filter, under conditions with the scalp and skull intact (i) and with the scalp removed (ii). The plots show a significant change in blood flow due to the application of the filter, particularly evident when the scalp and skull are intact. A slight significant change can also be observed with the scalp removed by applying the proposed filter. This highlights the importance and efficacy of the filter specifically in conditions with scalp and skull intact.

## 4 Discussion

In this study, we have developed and validated a modular imaging platform tailored for small animals, capable of quantifying both deep tissue and superficial cerebral blood flow. We demonstrated the platform’s efficacy in detecting relative blood flow changes through functional studies across the olfactory bulbs and somatosensory cortex in mice and rats. In addition, we have shown topographic changes in cerebral blood flow even with scalp and skull intact, by employing a novel topographic imaging method equipped with a filter and combined with M-DCT.

The modular design of the platform allows for independent operation of each imaging method, providing flexibility and customization to suit diverse experimental needs. The platform also facilitates seamless interaction between modules and real-time monitoring of animal health controlled by a user-friendly GUI and SBC. Our setup, as shown in Fig. 1, features precise positioning mechanisms for optical components and a customizable stereotaxic frame for placing animals, ensuring reproducibility and ease of use. This optimized and integrative design has enabled us to overcome several limitations associated with an ad hoc system design, such as ROI mismatch, inconsistencies in the implementation of functional stimulus (needle placement during forepaw stimulation or odor delivery during olfactory stimulation), and procedural delays during alignment contributing to animal deaths. This enables us to achieve more robust, reliable, and repeatable measurements with the proposed system. Our filter design effectively removes surface artifacts and improves the accuracy of blood flow measurements even with the scalp and skull intact as shown in Figs. 7 and 8. These results are in accordance with Refs. 50 and 53 which have reported CBF changes with forepaw stimulation in small animals using speckle-based methods. For implementing the filter, we have chosen the thickness of the scalp and skull in animals based on the literature;^38^^,^^39^ however, it is more appropriate to extract the information from CT/MRI. This modular design approach, as opposed to conventional ad hoc methods, combined with a topographic algorithm, will be beneficial to the small animal imaging community, particularly those employing speckle-based perfusion imaging techniques.

The application of our imaging platform to perform functional studies on the olfactory bulb of mice represents a new exploration into blood flow dynamics in response to different odors and sensory stimulation. The results in Fig. 5 show a positive frequency shift post-odor stimulation with the shift following a decreasing trend from AA to Cin. This matches the similar trend observed in Fig. 3(f) where the mean rCBF decreases from AA to Cin. These changes in the measured blood flow could be correlated to the pulse oximeter reading which showed an increase in breathing rate. The prior works^33^^,^^54^ have shown an increase in sniffing rate during odor stimulation in mice. Therefore, increase in blood flow rate could be attributed to the increase in sniffing rate during odor stimulation in mice. It must be noted that a significant frequency shift (p<0.05) was obtained for few odors. The system’s responsiveness to other odors could be improved by the selection of optimal exposure times of the camera for any odor. Despite limited reports,^55^^,^^56^ many questions remain unanswered on the contribution of vascular pulsations in driving the synchronized neuronal activity.^29^^,^^57^^,^^58^ In view of the efficiency we obtained in quantifying OB hemodynamics, our method can be further developed to study neurovascular coupling.

We note that the ICT values obtained from single-exposure LSCI in Figs. 3(e) and 4 are lower than expected by approximately an order of magnitude; however, this estimate improved and aligned with expected values when MESI data [Figs. 3(b) and 3(c)] were used.^46^ Nonetheless, the error associated with single exposure-based flow measurements is not a major concern for the present study as we report mostly the rCBF associated with the functional activations. We also note that the odor delivery system can be further improved by implementing complete automation. This will help us in reproducing the quantification of hemodynamic changes toward multiple odors. We have given enough time among trials along with the usage of exhaust airflow which is inbuilt in our imaging platform. The spatio-temporal series of plot for 60 s as shown in Fig. 4 helps to accurately localize the brain regions involved in processing smells and analyze how quickly these regions react over time. For the topographic studies of olfactory stimulation, we have performed both skull-intact and scalp-intact conditions to prove the compatibility of our instrument and method under different experimental conditions. Our system thus offers excellent spatial and temporal resolution, in a cost-effective manner, for quantification of blood flow with the possibility to see global changes with the skull and scalp intact. Our system, with the current collection optics, offers a spatial resolution of 150  μm for LSCI and 1 mm for topography. The data acquisition time for a single exposure raw intensity image is 4.3 ms. The full frame acquisition time is 80 s for multi-exposure LSCI data (200 frames, 10 exposure) and 760 s for nine-point topography study.

In comparison with existing methods employed in mouse olfactory studies, LSCI offers the advantage of real-time visualization of blood flow changes within vessels over a larger region. It effectively balances spatial and temporal resolution, rendering it well-suited for recording the dynamic reactions to olfactory stimulation. The combination of topographic scanning with the filter method offers an improved and faster reconstruction of blood flow in small animals. We note that the data acquisition through cameras and subsequent processing of speckle contrast and blood flow measurements using methods such as M-DCT could be time-consuming and adopting high-frame-rate cameras such as Single Photon Avalanche Diode (SPAD) arrays along with high-speed Graphical Processing Unit (GPU) or parallel processing systems can be used for faster computation. Also, the speed of processing can be further improved using better SBC.

We would like to stress the complementary perfusion measurements provided by LSCI and topographic scanning as a feature of a multi-modal imaging system. Although LSCI provides the superficial perfusion, topographic scanning with a filter removes surface artifacts and gives a projection of the deep tissue flow onto the XY plane (brain surface) which combined gives a pseudo-three-dimensional image of the blood flow. In the future, studies correlating blood flow changes to neural and behavioral responses toward different odorants and pheromones could be carried out, which will provide insights into information processing through olfactory subsystems.^59^

## 5 Conclusion

We have presented a laser speckle–based imaging platform and algorithms for imaging superficial and deep tissue cerebral blood flow in small rodents. We have also introduced a topographic imaging method with a filter to image cerebral blood flow in animals with scalp and skull intact, along with the modular design of the imaging platform which incorporates both LSCI and M-DCT. We have validated the effectiveness of the system through functional studies by detecting enhanced blood flow changes in the olfactory bulbs and somatosensory cortex, thus paving the way for more comprehensive studies in small animals using this system.

## Data Availability

The code and data utilized in this study are available from the authors upon reasonable request.
